# Tumor markers for hepatocellular carcinoma

**DOI:** 10.3892/mco.2013.119

**Published:** 2013-05-13

**Authors:** YAN-JIE ZHAO, QIANG JU, GUAN-CHENG LI

**Affiliations:** Tumor Immunobiology Laboratory of Cancer Research Institute, Key Laboratory of Carcinogenesis and Cancer Invasion Ministry of Education, Key Laboratory of Carcinogenesis Ministry of Health, Central South University, Changsha, Hunan 410078, P.R. China

**Keywords:** hepatocellular carcinoma, early diagnosis, tumor markers

## Abstract

Hepatocellular carcinoma (HCC) is one of the most common malignant tumors with a high rate of morbidity and mortality. HCC affects approximately one million individuals annually worldwide, with the incidence equal to the mortality rate. In 2008, HCC was listed as the third most lethal cancer. Thus, early diagnosis is crucial for improving the survival rate for patients. α-fetoprotein (AFP) together with iconography and pathology detection are commonly used in the clinical early diagnosis of liver cancer. However, the specificity and sensitivity of AFP used in screening for liver cancer are not satisfactory. Athough the development of molecular biology has led to the identification of new tumor markers, including proteantigens, cytokines, enzymes and isoenzymes, as well as related genes that can be used in the treatment and prognosis of liver cancer, more tumor markers are required for effective early diagnosis of diseases and monitoring of the curative effect.

## Contents

1. Introduction
2. Embryonic antigen
3. Proteantigen
4. Enzymes and isozymes
5. Cytokines
6. Genetic biomarkers
7. Conclusions

## 1. Introduction

Primary hepatic cancer (PHC) is one of the most common malignant tumors with 90–95% of liver cancer being hepatocellular carcinoma (HCC). Liver cancer exhibits no symptoms in the early stage, whereas clinical symptoms are evident in the advanced stage, leading to an unsatisfactory curative effect. In 2008, HCC was listed as the third most lethal cancer type (1). Findings of a previous study suggested that early diagnosis of HCC and effective treatment are likely to prolong the lifetime of liver cancer patients (2). Thus, the identification of new high sensitivity and specificity markers for HCC are essential.

α-fetoprotein (AFP) together with iconography and pathology detection are commonly used in clinical early diagnosis for liver cancer. However, the widely used marker AFP does not yield satisfactory results in the early diagnosis of HCC, particularly AFP-negative HCC. These false-negative results limit the universality of its application. In recent years, the development of molecular biology has led to the successful exploration and identification of markers for HCC, which is expected to improve the early diagnostic rate, treatment effect in addition to curative satisfaction.

## 2. Embryonic antigen

### AFP

AFP is the most widely used tumor biomarker currently available for the early detection of HCC. Findings of a previous clinical study demonstrated that serum AFP had a sensitivity of 41–65% and specificity of 80–94% when the cut-off value is 20 ng/ml (3). However, the following issues should be considered with regard to early diagnosis: i) the positive rate of AFP in HCC is only 60–80%, making it a limitation; ii) false-positive, AFP results are positive during pregnancy, as well as for active liver disease, embryonic tumor and certain gastrointestinal tumors; iii) false-negative, limitations in terms of sensitivity in different detection methods. Additionally, a small hepatic tumor results in AFP expression being lower than the limit of detection, whereas AFP expression is delayed or higher than the limit of detection when the tumor is large, yielding AFP-negative HCC.

### AFP heterogeneity

The development and applications of biological chemistry and related analysis, as well as additional study of AFP have revealed that AFP has three glycoforms (AFP-L1, AFP-L2 and AFP-L3), according to their binding ability to the lectin lens agglutinin (LCA). AFP-L1, as a non-LCA-bound heterogeneity, is a major glycoform in various benign liver diseases. By contrast, AFP-L3, as an LCA-bound heterogeneity, exists only in the serum of patients with HCC at a cut-off value of 15%, with a sensitivity and specificity of 96.9 and 92%, respectively, in detecting HCC (4). The sensitivity and specificity of AFP-l3 are both relatively satisfactory as compared with AFP. Moreover, AFP-L3 does not correlate with AFP, thus the former can be used as an independent and significant factor for the early diagnosis of HCC. Specifically, for HCC patients with a total AFP of 10–200 ng/ml, when the cut-off value of AFP-L3 is 35%, the diagnostic specificity for HCC reaches 100%, thereby improving the early diagnostic rate (5). Using the *μ*TAS method, ALP-L3% was detected with high sensitivity in the diagnosis of HCC at stage I or when the tumor size was <2 cm (42.5 and 46.0%, respectively). When the cut-off value of AFP-L3% is 5%, the sensitivity for HCC reaches 47.2% compared with that of 38% for total AFP (6). Consequently, AFP-L3 is expected to be a useful marker for HCC.

## 3. Proteantigen

### HSP

Heat shock protein (HSP) is a highly conserved stress response protein. It can protect cells and promote them to repair the damage caused by a variety of stimuli. HSP is expressed under physiological and stress conditions, including carcinogenesis. By immunohistochemical staining, Joo *et al* (7) identified the positive rate of HSP70 and HSP27 to be 56.3 and 61.9%, respectively, in HCC tissues. The stained intensity of HSP70 was positively correlated with tumor size, portal vein invasion and tumor stage, while HSP27 was only associated with HCC which are infected by hepatitis B virus (HBV). In the microenvironment of hepatocarcinoma, the overexpression of HSP70 and HSP27 was able to promote tumor growth and metastasis (8).

HSP70 may be used as an indicator of prognosis for HCC. Its expression was observed in 282 of 392 HCC cases (71.9%), whereas only 14 of 115 non-neoplastic liver tissues expressed HSP70 (P<0.001) (9). The sensitivity and specificity in detecting HCC were identified as 57.5 and 85%, respectively (10). Furthermore, the expression of HSP70 is correlated with differentiation and apoptosis of tumor cells. It promotes tumor cell growth by stabilizing cyclin D1 and suppresses the apoptosis of tumor cells by inhibiting the p53 pathway (11,12). Thus, HSP70 and HSP27 are potential markers for HCC and should be further investigated.

### Glypican-3 (GPC3)

GPC3 is a family of the heparan sulfate proteoglycans that is linked to the cell membrane by a glycosylphosphatidylinositol (GPI) anchor (13). GPC3 is involved in the process of regulating cell growth, development, differentiation and migration. sGPC3, which lack GPI, inhibit the growth of HCC by removing several pro-tumorigenic growth factors including Wnts, hepatocyte growth factor (HGF) and vascular endothelial growth factor (VEGF) from the surface of HCC cells *in vitro* and *in vivo*(14). The expression of GPC3 was upregulated in HCC tumor tissues compared with normal and benign liver diseases and contributed to promoting the growth of HCC by stimulating Wnt signaling (15). In addition, no correlation between GPC3 expression and tumor stage, size and AFP level has been observed. The sensitivity and specificity in the diagnosis of HCC was found to be 77 and 96%, respectively (16). On the strength of these results, GPC3 is a potential marker for HCC.

### SCCA

Squamous cell carcinoma antigen (SCCA), a serine protease inhibitor isolated from cervical carcinoma, is typically expressed in epithelial tumors and protects tumor cells from apoptosis. Guido *et al* found that the expression of SCCA in HCC (93%) and dysplastic nodule (100%) is significantly higher than the regenerative nodule (29%), suggesting that the expression of SCCA increased in the early stages of HCC formation (17). In addition, high SCCA values in the tumor were inversely correlated with the nodule size (18), which had a sensitivity and specificity of 84 and 46%, respectively, in detecting HCC (19). The high sensitivity and low specificity were complementary with AFP. Thus, SCCA was a valuable supplement marker for the diagnosis of HCC.

SCCA-IgM IC is a circulating immune complex composed of SCCA and IgM. It was undetectable in the sera of a healthy control population. However, in chronic hepatitis, cirrhosis and HCC, the detection rates of SCCA-IgM IC were 18, 26 and 70%, respectively. No correlation was identified with AFP level (20). Furthermore, in patients with LC progressing towards HCC, SCCA-IgM IC was consistently increased and had higher sensitivity compared with AFP (21). Therefore, SCCA-IgM IC may be a novel valuable serum marker for HCC. A combination of SCCA-IgM IC and AFP can thus improve the diagnostic rate.

### Golgi protein 73 (GP73)

GP73 (also known as Golph2 and GOLM1) is a type II Golgi-specific membrane protein that is coded by the GOLM1 gene on chromosome 9q21.33 (22). It is significantly elevated in various types of cancer, such as lung adenocarcinoma (23), seminomas (24) and renal cell cancer (25). However, GP73, is closely associated with liver diseases, particularly HCC, and has received increasing attention. Results of recent studies have shown that the serum GP73 is significantly elevated in primary hepatic carcinoma (PHC) (26,27). In liver cirrhosis it is not only elevated, but also higher than in HCC, whether infection is caused by HBV or hepatitis C virus (HCV) (28). By contrast, in normal liver, GP73 is expressed by biliary epithelial cells, but minimally by hepatocytes (29). Thus, GP73 is a significant factor in many hepatic diseases. In their study, Mao *et al* (31) demonstrated that GP73 in the serum of patients with HCC infected by HBV was significantly higher compared with HBV carriers, patients without hepatic diseases and healthy adults. The sensitivity of diagnosis of HCC (76.9%) was markedly elevated compared with AFP (48.6%), suggesting GP73 is a novel and effective serum biomarker for the diagnosis of HCC (30,31).

Additional investigations identified fucosylated GP73 (FC-GP73). Compared with total GP73, FC-GP73 improves the sensitivity and specificity of diagnosis of HCC from 65–90 to 90–100%, respectively. For GP73-negative or low levels, detection of FC-GP73 is a viable option (32). Although the study for GP73 is optimistic, there are limitations that should be considered such as the fact that the correlation between GP73 and tumor size, stage, recurrence and prognosis should be extensively investigated. Specifically, the mechanism for GP73 and HCC development remains to be elucidated. Thus, role of GP73 in the clinic remains to be determined.

### Tumor-associated glycoprotein 72 (TAG-72)

TAG-72 is a macro-molecular glycoprotein complex similar to mucin-1 (MUC-1). It is overexpressed in the majority of human adenocarcinomas including gastric, colon and pancreatic cancer. However, it is rarely expressed in normal tissues. Recent studies (33) found that the expression of TAG-72 is significantly elevated in HCC tissues compared with normal liver tissues. Its increased expression may promote tumor invasion and metastasis. Furthermore, overexpression of TAG-72 is closely correlated with poor survival in patients with HCC (33). Thus, TAG-72 is a potential prognostic marker for HCC, which has important clinical implications. Moreover, anti-TAG-72 monoclonal antibody has been used for the clinical detection of tumors (34).

### Zinc-α2-glycoprotein (ZAG)

ZAG, a soluble glycoprotein, is a member of the class I major histocompatibility complex (MHC-I) family. Due to its high homology of the amino acid sequence with the lipid mobilizing factor (LMF), we considered it a novel adipokine. ZAG is downregulated in human obesity (35). By contrast, it is upregulated in several types of cancer such as breast, lung and prostate cancer and is considered as a potential biomarker for these cancer types. By analyzing the serum proteome of the HCC group, liver cirrhosis group and healthy adults, we confirmed that the ZAG protein is overexpressed in the HCC group, suggesting it is a novel candidate biomarker for the early diagnosis of HCC (36).

## 4. Enzymes and isozymes

### Des-γ-carboxyprothrombin (DCP)

DCP is an abnormal protein induced by the absence of vitamin K. When the liver cells are in the process of malignant transformation, an obstacle occurs for the vitamin K-dependent carboxylation system. Insufficient reaction of γ-glutamyl carboxylation causes the production of DCP (37). The level of DCP in the serum of patients with HCC was significantly higher than that in patients with chronic hepatitis and cirrhosis. The diagnostic sensitivity of DCP was weaker compared with AFP when HCC was <3 cm in diameter, while it was stronger compared with AFP when HCC was >5 cm in diameter (38). However, Baek *et al* recently demonstrated that irrespective of whether the diameter of HCC is <3 cm, 3–5 cm or >5 cm, the diagnostic accuracy of DCP was higher than that of AFP (39). In addition, the combined detection of DCP and AFP can improve the diagnostic sensitivity and can be used to predict the recurrence of HCC within 6 months after surgery (40). By contrast, the level of DCP is closely associated with a larger tumor, vascular invasion and it serves as a more accurate tumor marker compared with AFP and AFP-L3 (41).

### γ-glutamyl transferase (GGT)

GGT, a plasma membrane-bound enzyme, is synthesized in the microsomes of human cells. It is highest in embryo livers and decreases rapidly to the lowest levels after birth (42). The activity of serum GGT is extremely low in healthy adults, but stimulations such as cholestasis and inflammation can improve the level of serum GGT to varying degrees. GGT mRNA is widely distributed in the liver tissues of healthy adults, patients with liver disease, benign liver tumor and HCC (4). The development of HCC due to cancer cell inverse differentiation is similar to the embryonic stage, improving the level of serum GGT to a moderate or high degree. Therefore, GGT may serve as a marker for HCC. However, the sensitivity of GGT to detect small HCC is only 43.8% (43). This enzyme is therefore only used to aid in the diagnosis of HCC. In addition, it has been previously shown that simultaneous determination of GGT, DCP and AFP can significantly improve the rate of diagnosis of HCC (44).

### α-l-fucosidase (AFU)

AFU is a lysosomal enzyme widely present in human cells, blood and body fluid. It can be detected in the serum of healthy adults, although its activity increases in the serum of HCC patients. Additionally, it does not correlate with tumor size or the level of AFP (45). It has been reported that the sensitivity, specificity and diagnostic accuracy of AFU at the cut-off value of 2.3005 *μ*mol/l/min were 90, 97.5 and 94.9%, respectively. The combined detection of AFP and AFU can improve the sensitivity, specificity and diagnostic accuracy of AFP from 70, 85 and 79.7%, respectively, to 95, 100 and 99.1%, respectively (46). In addition, AFU can reveal the case 6–9 months earlier than ultrasonographic visualization (47). Thus, AFU may be a valuable supplementary marker in HCC detection. However, the specificity of AFU is relatively poor, and is also overexpressed in diabetes, pancreatitis and hypothyroidism patients. The activity of AFU is also susceptible to ethnicity. Therefore, the clinical value of AFU requires additional investigation.

## 5. Cytokines

### Transforming growth factor-β1 (TGF-β1)

TGF-β1 is a pluripotent growth factor involved in the regulation of cell proliferation, differentiation, embryo formation, angiogenesis, invasion and immune function. It is highly expressed in tumor cells, inhibiting the proliferation of tumor specific cytotoxic T lymphocytes (CTL) and NK cells, promoting the growth of tumor cells. The level of serum TGF-β1 has been reported to be elevated in HCC patients compared with healthy adults or patients with non-malignant liver disease (48). It could induce microvascular abnormalities through the downregulation of neural cell adhesion molecules in human HCC (49). TGF-β1 and TGF-β1 mRNA may be used as sensitive indicators to diagnose HCC which is induced by HBV, with the sensitivity and specificity being 89.5 and 94.0%, respectively, when TGF-β1 is >1.2 *μ*g/l (50). The study of TGF-β1 demonstrated that TGF-β1 mediated different biological effects through different signaling pathways. The polymorphism of TGF-β1 expression can affect susceptibility to tumor. TGF-β1 signaling pathway as a tumor therapeutic target has become a hot spot.

### VEGF

VEGF is well known to play a crucial role in tumor angiogenesis by inducing new vessel formation and promoting tumor invasion and metastasis. The expression of VEGF is higher in HCC patients. Xiang *et al* (51) revealed that VEGF is a type of biomarker of lymph node metastasis in HCC. In addition, the expression of VEGF is closely correlated with tumor recurrence and prognosis. Of note, some VEGF receptor expression has been found to correlate with the development of tumor (52). Knockdown of VEGF165 is able to inhibit the proliferation, migration and adhesion ability of HCC cells by increasing the expression of p53 signaling molecules (52). Therefore, the overexpression of VEGF may be useful as a biologic marker of tumor invasiveness and in predicting poor prognosis.

## 6. Genetic biomarkers

### AFP mRNA

AFP mRNA is considered the most valuable marker for circulating HCC cells and is only present in active HCC cells. If other interferences such as genital tumors and peripheral blood are excluded, AFP mRNA could be used as a significant marker for spreading of HCC in blood. In advanced liver cancer, the rate of AFP mRNA expression reaches 100%. Moreover, the value of AFP mRNA serves as a predictor for HCC recurrence. However, use of this marker is controversial, possibly due to the blood-borne dispersion of normal liver cells and tumor cells and the mis-transcription of mRNA encoding AFP by peripheral mononuclear cells (4). The positive rate of AFP mRNA in the recrudescent patients was 82.4%, significantly higher compared with the group without recurrence (53). Thus, AFP mRNA effectively predicts tumor recurrence and metastasis following surgery.

### MicroRNAs

MicroRNAs are small non-coding RNAs that effectively block translation by promoting the degradation of target mRNAs or binding to complementary sequences in the 3′ untranslated region (UTR). In recent years, the association between microRNAs and tumors has becomes a point of debate. MiR-500 (miRNA) is a potential candidate biomarker for HCC, as proven by Yamamoto *et al* (54), using a global miRNA expression profile in mouse liver development. This profile was highly expressed in embryo liver, downregulated in the process of liver development and then upregulated in the process of cirrhosis. Based on miRNA microarray, miR-29 was downregulated in HCC cells, suggesting its role as a prognostic marker for HCC therapy (55). Moreover, miR-199a/b-3p was also downregulated in HCC compared with normal liver and hepatitis liver, indicating its role as a prognostic target for HCC (56). MiR-122 is a liver-specific microRNA that is downregulated in HCC cells. Overexpression of miR-122 maintained cells in G2/M phase by regulating MDR expression (57). However, loss of miR-122 promoted HCC cell migration and invasion, rendering miR-122 a prognostic target for HCC.

An analysis of the miRNA signatures of a large number of tumor samples revealed that only miR-21 is upregulated in the tumors (58). Moreover, similar results have demonstrated that miR-21 is a central oncomiR (59). Plasma miR-21 level in patients with HCC was significantly higher than that in patients with chronic hepatitis and healthy individuals. It promoted HCC growth by regulating the expression of PENT and PENT-related pathways (60). ROC analysis revealed the sensitivity and specificity to be 87.3 and 92%, respectively, differentiating HCC patients from healthy adults. Thus, miR-21 is also a promising biomarker of HCC (61).

### Related genes

Δ-like 1 homolog (DLK1) is a newly identified hepatic stem/progenitor cell marker in fetal livers which is crucial in the oncogenesis of HCC. DLK1 is usually only expressed in embryonic, kidney and neuronal tissue. However, in HCC, DLK1 expression is significantly elevated and a similar phenomenon was also observed in small cell lung cancer, neuroblastoma and leukemia, suggesting that DLK1 is important in the occurrence of these tumors. Jin *et al* (62) reported that DLK1 expression does not correlate with AFP level and tumor metastasis, suggested DLK1 is an independent prognostic marker (62).

Hepatoma-associated gene (HTA) is a tumor differentially expressed gene obtained by bioinformatic analysis. Findings of a recent study showed that HTA is present only in tumors and absent in normal tissues. It is tumor-specific and the positive rate is extremely high in HCC in particular, suggesting a key role in the diagnosis and treatment of HCC (63).

Villin1 (Vil1) is a newly identified marker for HCC. The upregulation of Villin1 mRNA in high serum AFP-associated HCC tumor tissues induced poor differentiation, vascular invasion, advanced cancer stage and recurrence-free survival. Thus, Vil1 is a potential candidate molecular marker for high serum AFP-associated HCC (64).

## 7. Conclusions

Tumor progression is a complex disease process involving various factors, changes of various genes, and the development of multiple stages. During tumor progression, the number, species, distribution and expression level of tumor markers in patients with HCC exhibit variations that are closely associated with the occurrence, development, transfer, effect and prognosis of tumors. Large numbers of HCC markers exist in the clinical setting, however, most single indicators lack specificity of the tissues and organs. Furthermore, the single indicator results in the varying degrees of false positivity in certain benign diseases. Therefore, effective test strategies should be considered to improve the early diagnostic rate of HCC including: i) the combined detection of several serum markers that can complement each other in order to improve the early diagnostic rate. The sensitivity of AFP-L3, FC-GP73, AFU and SCCA is superior to that of AFP (Table I). Combined detection can increase the rate of diagnosis of HCC and reduce misdiagnosis. Other markers including GPC3 and TGF-β1 have a very high specificity. Combined detection with AFP can significantly improve the ability of identification and diagnosis for HCC. ii) Selection of different indices based on different detection purposes: for example, AFP mRNA, TGF-β1 and VEGF should be the first option in the prediction of HCC metastasis. iii) Diagnosis of HCC at the genetic level through the detection of the expression of AFP mRNA in peripheral blood, which is likely to improve the early diagnostic rate of HCC, as well as predict the transfer and recurrence of tumors. iv) The diagnosis of HCC must be combined with clinical manifestation, iconography detection and histological examination. Additional studies are likely to yield novel markers and adopt more effective combined detection methods to improve the sensitivity and specificity of diagnosis of HCC, resulting in improved treatment and prognosis.

This study was supported by the National Natural Science Foundation of China (nos. 81201903 and 2010CB833605), the Science and Technology Department Research Foundation of Hunan (2010SK3132) and the Ph.D. candidates' research innovation project of Hunan (CX2011B050), China.

## Figures and Tables

**Table I:** Markers for HCC.

Markers	The expression in the serum/tissues of HCC	Sensitivity (%)	Specificity (%)	Application
AFP	Upregulation	41.0–65.0	80.0–94.0	Early diagnosis
AFP-L3	Upregulation	96.9	92.0	Early diagnosis
HSP70	Upregulation	57.5	85.0	Prognosis
GPC3	Upregulation	77.0	96.0	Diagnosis
SCCA	Upregulation	84.0	46.0	Early diagnosis
GP73	Upregulation	76.9	-	Diagnosis
FC-GP73	Upregulation	90.0	100.0	Diagnosis
GGT	Upregulation	43.8	-	Diagnosis
AFU	Upregulation	90.0	97.5	Diagnosis
AFU+AFP	Upregulation	95.0	100.0	Diagnosis
TGF-β1	Upregulation	89.5	94.0	Prognosis
VEGF	Upregulation	-	-	Recurrence and prognosis
AFP-mRNA	Upregulation	-	-	Recurrence and prognosis
miR-21	Upregulation	87.3	92	Diagnosis
miR-500	Downregulation	-	-	Prognosis
miR-29	Downregulation	-	-	Prognosis
miR-122	Downregulation	-	-	Prognosis

HCC, hepatocellular carcinoma; AFP, α-fetoprotein; HSP70, heat shock proteins 70; GPC3, Glypican-3; SCCA, squamous cell carcinoma antigen; FC-GP73, fucosylated GP73; GGT; γ-glutamyl transferase; AFU, α-l-fucosidase; TGF-β1, transforming growth factor-β1; VEGF, endothelial growth factor; miR, miRNA.
